# Optimization of HU threshold for coronary artery calcium scans reconstructed at 0.5‐mm slice thickness using iterative reconstruction

**DOI:** 10.1002/acm2.12806

**Published:** 2019-12-30

**Authors:** Kuei‐Yuan Hou, Katsumi Tsujioka, Ching‐Ching Yang

**Affiliations:** ^1^ Department of Radiology Cathay General Hospital Taipei Taiwan; ^2^ Faculty of Radiological Technology Fujita Health University Aichi Japan; ^3^ Department of Medical Imaging and Radiological Sciences Kaohsiung Medical University Kaohsiung Taiwan

**Keywords:** Agatston score, coronary artery calcification, iterative reconstruction, protocol optimization

## Abstract

**Purpose:**

This work investigated the simultaneous influence of tube voltage, tube current, body size, and HU threshold on calcium scoring reconstructed at 0.5‐mm slice thickness using iterative reconstruction (IR) through multivariate analysis. Regression results were used to optimize the HU threshold to calibrate the resulting Agatston scores to be consistent with those obtained from the conventional protocol.

**Methods:**

A thorax phantom set simulating three different body sizes was used in this study. A total of 14 coronary artery calcium (CAC) protocols were studied, including 1 conventional protocol reconstructed at 3‐mm slice thickness, 1 FBP protocol, and 12 statistical IR protocols (3 kVp values*4 SD values) reconstructed at 0.5‐mm slice thickness. Three HU thresholds were applied for calcium identification, including 130, 150, and 170 HU. A multiple linear regression method was used to analyze the impact of kVp, SD, body size, and HU threshold on the Agatston scores of three calcification densities for IR‐reconstructed CAC scans acquired with 0.5‐mm slice thickness.

**Results:**

Each regression relationship has R^2^ larger than 0.80, indicating a good fit to the data. Based on the regression models, the HU thresholds as a function of SD estimated to ensure the quantification accuracy of calcium scores for 120‐, 100‐, and 80‐kVp CAC scans reconstructed at 0.5‐mm slice thickness using IR for three different body sizes were proposed. Our results indicate that the HU threshold should be adjusted according to the imaging condition, whereas a 130‐HU threshold is appropriate for 120‐kVp CAC scans acquired with SD = 55 for body size of 24.5 cm.

**Conclusion:**

The optimized HU thresholds were proposed for CAC scans reconstructed at 0.5‐mm slice thickness using IR. Our study results may provide a potential strategy to improve the reliability of calcium scoring by reducing partial volume effect while keeping radiation dose as low as reasonably achievable.

## 1 INTRODUCTION

Coronary artery disease (CAD) is one of the leading causes of death around the world. Meanwhile, atherosclerosis is the most common form of vascular disease and constitutes the major cause of death, with 17.5 million global deaths annually.^1^ The hallmark lesion in atherosclerosis is the atherosclerotic plaque, whereas rupture‐prone atherosclerotic plaques, that is, vulnerable plaques, may cause life‐threatening events like acute coronary syndrome or stroke. In general, a vulnerable plaque is often found to be associated with a thin fibrous cap, a high inflammation burden, a large lipid pool, macroscopic heterogeneity, and so on. Spotty calcification is a morphological characteristic of a vulnerable plaque phenotype.^2^

Coronary artery calcium (CAC) examination performed on computed tomography (CT) is widely considered as a reliable technique for screening risk of future cardiac events.^3^ The reference protocol for CAC acquisition has a tube voltage of 120 kVp, and scan data are reconstructed at 3‐mm slice thickness with filtered back‐projection (FBP). The threshold for the determination of coronary calcification is 130 Hounsfield unit (HU). The amount of calcium is typically quantified with the Agatston score, which is a function of the CAC area and the maximal CAC density.^4^, ^5^, ^6^ The total Agatston score is the sum of each CAC lesion for all coronary arteries extending through the z‐axis of the heart.^7^ A reduced slice thickness reduces partial volume artifacts, possibly increasing reproducibility of CAC scoring and decreasing overestimation of fully calcified plaques. But this would make the score more susceptible to noise. van der Werf et al. reported that high image noise would increase coronary artery scores.^8^ According to their multivendor phantom study, Agatston score increased by 1–8% at 40% reduced dose and 23–64% at 80% reduced dose. On the other hand, Willemink et al.^9^ and Li et al.^10^ reported that high image noise would decrease coronary artery scores. Depending on the vendor, this underestimation can be up to 50%.

This study aimed to improve the reliability of calcium scoring by using 0.5‐mm slice thickness to reduce partial volume effect while keeping radiation dose as low as reasonably achievable (ALARA). CT radiation dose reduction is frequently achieved by applying lower kVp^11^, ^12^, ^13^ and tube current modulation (TCM).^14^, ^15^, ^16^ Iterative reconstruction (IR) methods are developed to reduce image noise by performing a recursive search for the best estimate, so they can provide a similar image quality at higher noise levels compared with FBP. Consequently, applying IR techniques is considered to be one of the strategies for reducing radiation dose in CT.^17^, ^18^, ^19^, ^20^ However, these dose reduction strategies would affect the image performance of CT in terms of contrast, spatial resolution, and noise pattern, and thus may impact the CAC scores. Therefore, this work first investigated the influence of kVp, mA, and HU threshold on calcium scoring for CAC scans reconstructed at 0.5‐mm slice thickness using IR through anthropomorphic phantom studies. Based on these study results, the optimized HU thresholds were then proposed for CAC scans under various imaging conditions to calibrate the resulting Agatston scores to be consistent with those obtained from reference protocol.

## 2 METHODS

### 2.1 Anthropomorphic chest phantom

**Figure 1:**
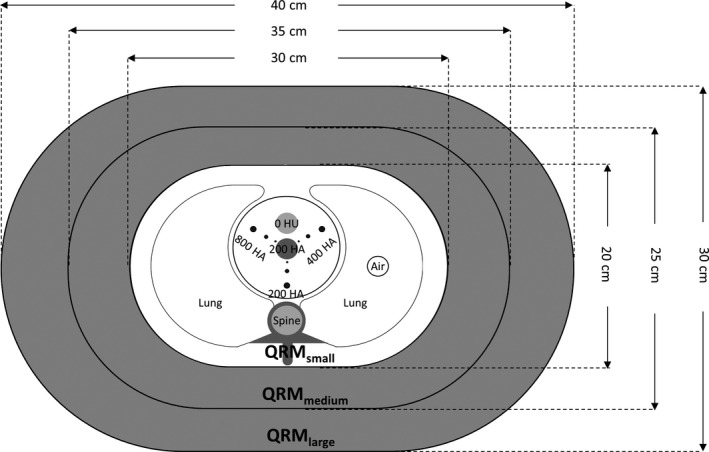
Illustration of the anthropomorphic chest phantom (HA: calcium hydroxyapatite; HU: Hounsfield unit).

A thorax phantom (Thorax‐CCI, QRM GmbH, Möhrendorf, Germany) which consists of an anthropomorphic phantom body and a calibration insert was used for CAC scans (Fig. 1). The anthropomorphic phantom body contains artificial lungs and a spine insert surrounded by tissue equivalent material. The outer dimensions of the phantom body are 300 × 200 mm^2^ in transverse plane and 150 mm in height (QRM_small_). Two extension rings were added to the phantom body to enlarge the phantom size in transverse plane to 350 × 250 mm^2^ (QRM_medium_) and 400 × 300 mm^2^ (QRM_large_). The calibration insert contains nine cylindrical calcifications of three sizes (1, 3, and 5 mm in diameter and height) and three calcium hydroxyapatite (HA) densities (200, 400, and 800 mg/cm^3^). In addition, the calibration insert contains two large homogeneous inserts made of water equivalent material and HA with density of 200 mg/cm^3^. A cardiac simulator (Cardiac Trigger, Model: CTM300, Ivy Biomedical Systems, Branford, CT, USA) was used to synthesize ECG signal at mean heart rate of 60 bpm.

## 3 CAC SCANS

All CAC scans were performed on a 320‐detector row CT system (Aquilion One, Canon, Otawara, Japan) using a 0.35 seconds of gantry rotation time and half‐scan reconstruction. In our department, the routine CAC scans are acquired using TCM (SureExposure 3D, Canon, Otawara, Japan) at 120 kVp with 3‐mm slice thickness and reconstructed by FBP with soft tissue kernel (FC12). The TCM parameter value used to specify the desired level of image noise is the SD value, which is the standard deviation of HU in the central region of an image of a homogeneous water phantom. Besides the SD value, the TCM system allows the operator to define the mA range (minimum‐to‐maximum mA) within which the tube current can be modulated. The TCM system is operated with SD = 55 and mA range 40–300 mA in our routine clinical practice. A total of 14 CAC protocols of various acquisition/reconstruction combinations were studied, including 2 protocols with FBP reconstruction and 12 protocols with IR reconstruction. Table 1 demonstrates the scan parameters and the corresponding volume CT dose index (CTDI_vol_) displayed on the scanner console of the 14 CAC protocols investigated in this study. For the FBP group, CAC scans were acquired using two different slice thickness (3, 0.5 mm) and TCM with SD = 55. The tube voltage applied in the FBP group was 120 kVp. As for the IR group, CAC scans were acquired using three different tube voltages (120, 100, and 80 kVp) and TCM with four different SD values (55, 75, 115, and 155). The slice thickness applied in the IR group was 0.5 mm, and the reconstruction technique was Adaptive Iterative Dose Reduction 3D (AIDR 3D) at the standard level with FC12 kernel. All CAC scans employed a 22‐cm display field of view.

**Table 1:** The scan parameters, radiation dose and CT number within background ROI (mean ± standard deviation) of the phantom studies.

Reconstruction algorithm & slice thickness	Tube voltage & TCM parameters	QRM_small_			QRM_medium_			QRM_large_		
		Tube current (mA)	CTDI_vol_ (mGy)	CT number (HU)	Tube current (mA)	CTDI_vol_ (mGy)	CT number (HU)	Tube current (mA)	CTDI_vol_ (mGy)	CT number (HU)
FBP, 3 mm	120 kVp, SD = 55 (40–300 mA)*	40	1.1	41.43 ± 27.52	50	1.4	40.11 ± 41.47	150	4.2	40.60 ± 40.69
FBP, 0.5 mm	120 kVp, SD = 55 (40‐580 mA)*	60	1.7	41.86 ± 49.17	180	5.0	40.16 ± 47.04	520	15.6	40.58 ± 45.52
AIDR, 0.5 mm	120 kVp, SD = 55 (40–580 mA)*	60	1.7	45.51 ± 25.32	170	4.8	45.27 ± 24.26	510	15.3	48.00 ± 23.97
	120 kVp, SD = 75 (40–580 mA)*	40	1.1	40.60 ± 28.33	100	2.8	41.64 ± 26.66	290	8.7	40.69 ± 28.13
	120 kVp, SD = 115 (40–580 mA)*	40	1.1	41.11 ± 27.51	45	1.3	41.22 ± 32.92	130	3.6	39.25 ± 34.58
	120 kVp, SD = 155 (40–580 mA)*	40	1.1	41.70 ± 27.61	40	1.1	40.28 ± 36.38	70	2.0	39.10 ± 35.47
	100 kVp, SD = 55 (40–580 mA)*	100	1.7	34.66 ± 24.33	300	3.6	34.73 ± 23.58	580	10.6	38.61 ± 25.38
	100 kVp, SD = 75 (40–580 mA)*	60	1.0	29.66 ± 26.48	170	2.9	30.54 ± 25.54	500	9.2	31.87 ± 26.27
	100 kVp, SD = 115 (40–580 mA)*	40	0.7	29.48 ± 30.03	80	1.4	31.39 ± 30.04	230	3.9	30.65 ± 30.87
	100 kVp, SD = 155 (40–580 mA)*	40	0.7	29.61 ± 27.55	45	0.8	30.28 ± 35.73	130	2.2	29.53 ± 30.63
	80 kVp, SD = 55 (40–580 mA)*	230	2.0	12.97 ± 26.07	580	5.4	15.95 ± 29.12	580	5.4	22.58 ± 38.22
	80 kVp, SD = 75 (40–580 mA)*	140	1.2	9.79 ± 32.49	440	4.1	11.88 ± 30.45	580	5.4	13.60 ± 39.35
	80 kVp, SD = 115 (40–580 mA)*	70	0.6	6.97 ± 38.39	210	1.8	10.73 ± 37.21	580	5.4	16.33 ± 36.92
	80 kVp, SD = 155 (40–580 mA)*	40	0.3	8.61 ± 38.86	130	1.1	13.75 ± 41.06	380	3.3	13.32 ± 41.04

## 4 IMAGE QUALITY ASSESSMENT

The effects of statistical fluctuation were assessed in both low‐ and high‐contrast objects. For low‐contrast object, a background region of interest (ROI) of 35 by 35 pixels was placed at the center of the large homogeneous insert that is made of water equivalent material to calculate the mean and standard deviation of HU within background ROI. As for high‐contrast object, histograms of 5‐mm calcifications with HA density of 200, 400, and 800 mg/cm^3^ were generated and fitted by a Gaussian distribution to investigate the intensity distribution of calcifications.^21^ Spatial resolution was quantified by determining the modulation transfer function (MTF) calculated using the edge technique.^22^ The edge spread function was determined by a square ROI placed over the boundaries (1) at soft tissue/lung interface and (2) between the large homogeneous insert with HA density of 200 mg/cm^3^ and the surrounding soft tissue. These image quality assessments were performed in MATLAB 7.1 (The Mathworks, Natick, MA, USA).

## 5 CAC SCORING

Evaluating calcium score was performed on the postprocessing workstation (Vitrea FX, Vital Images, Minnetonka, USA), using calcium score analysis software (VScore, Vital Images, Minnetonka, USA). The Agatston score was calculated for each calcified lesion by multiplying the area (A_i_) of the lesion by a weighting factor (w_i_), determined by the density within the insert:

(1) $$\text{Agatston score}=\sum_{\text{all ROIs}}w_{i}\times A_{i}$$

For the reference protocol, the minimum HU threshold to identify calcium is predefined at 130 HU. A weighting factor of 1 is applied if the maximum CT value is between 130 and 199 HU, a weighting factor of 2 for 200 to 299 HU, a weighting factor of 3 for 300 to 399 HU, and a weighting factor of 4 for values above 400 HU:

(2) $${\text{w}}_{\text{i}}=\left\{\begin{array}{c} \begin{array}{c} 1,\;\text{if}\;130\;\text{HU}\leq {\text{CT}}_{\max}<200\;\text{HU}\\ 2,\;\text{if}\;200\;\text{HU}\leq {\text{CT}}_{\max}<300\;\text{HU} \end{array}\\ \begin{array}{c} 3,\;\text{if}\;300\;\text{HU}\leq {\text{CT}}_{\max}<400\;\text{HU}\\ 4,\;\text{if}\;400\;\text{HU}\leq {\text{CT}}_{\max}<400\;\text{HU} \end{array} \end{array}\right.$$

where CT_max_ is the maximum CT number in the lesion. Calcified lesions are excluded from the Agatston score if they have an axial area less than 1 mm^2^ or a CT number less than the predefined CT number threshold, that is, 130 HU at 120 kVp. It has been reported that lower kVp settings would increase the CT number of calcifications, and thus may result in overestimation of Agatston score.^23^, ^24^ In this study, the CAC scans were acquired not at 120 kVp but also at 100 and 80 kVp. Hence, three different HU thresholds were applied to detect calcification for each CAC scan listed in Table 1, including 130, 150, and 170 HU.

## 6 MULTIVARIATE ANALYSIS

A multiple linear regression method was used to analyze the impact of (1) tube voltage, (2) SD value, (3) body size, and (4) HU threshold on Agatston score (AS) obtained from CAC scans reconstructed at 0.5‐mm slice thickness using IR. The model to explain the dependence relationship was defined as:

(3) $$\text{AS}=B_{0}+B_{1}\times \left(\frac{1}{\text{kVp}}\right)+B_{2}\times \left(\text{SD}\right)+B_{3}\times \left(\text{body}\;\text{size}\right)+B_{4}\times \left(\frac{1}{\text{threshold}}\right)$$

where B_0_ to B_4_ are the regression coefficient (B_i_) to be estimated, and body size is the square root of the product of long and short axis of chest phantom, that is, the effective diameter. The coefficient of determination (R^2^) was calculated to assess the overall strength of the functional regression model. The standard regression coefficient (β_i_) was calculated to assess the relative importance of each predictor (X_i_), that is, β_i_ is standardized B_i_. The student’s t ratio (t_i_), which is the ratio of B_i_ and its corresponding standard error, was used to evaluate the significance of each predictor. A predictor was considered statistically significant if *P* < 0.01. A variance inflation factor (VIF_i_) is computed for each predictor, using the following formula: VIF_i_ = 1/(1‐R_i_^2^), where R_i_^2^ is obtained from the multiple regression in which X_i_ is predicted from all the other independent variables. A maximal VIF_i_ value in excess of 10 was regarded as an indication that multicollinearity may be unduly influencing the least square estimates. The statistical analysis algorithms were implemented in MATLAB 7.1 (The Mathworks, Natick, MA, USA).

## 7 RESULTS

### 7.1 Image quality assessment

**Figure 2:**
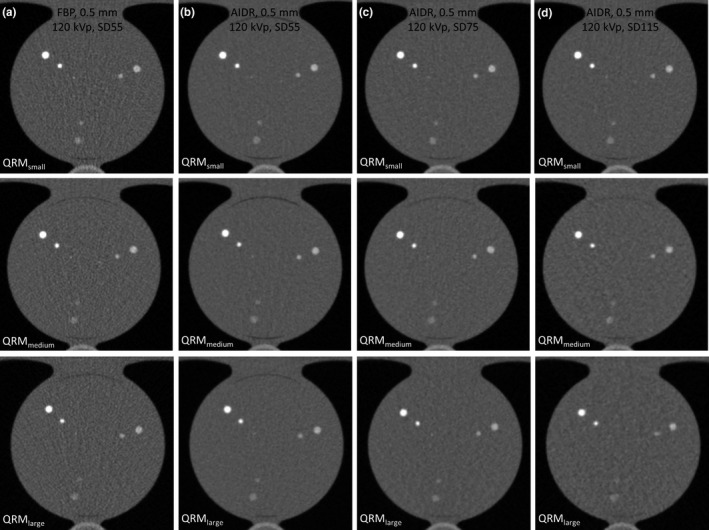
CT images acquired with four different acquisition/reconstruction combinations for QRM_small_ (top row), QRM_medium_ (mid row), and QRM_large_ (bottom row): (a) FBP, 0.5 mm, 120 kVp, SD = 55; (b) AIDR, 0.5 mm, 120 kVp, SD = 55; (c) AIDR, 0.5 mm, 120 kVp, SD = 75; and (d) AIDR, 0.5 mm, 120 kVp, SD = 115

**Figure 3:**
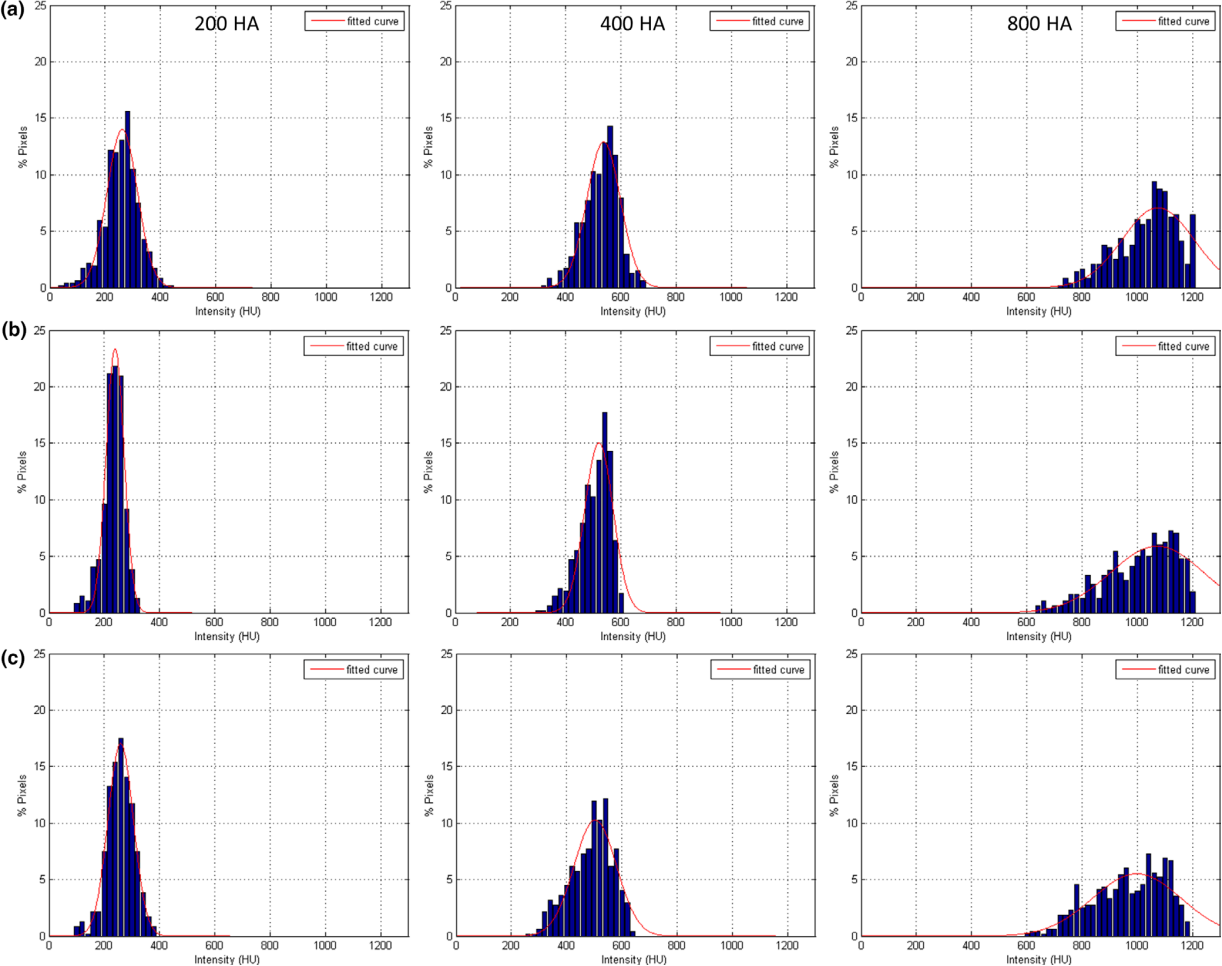
Histograms of calcifications with HA density of 200, 400, and 800 mg/cm^3^ (left to right) in CT scans acquired with three different acquisition/reconstruction combinations for QRM_medium_: (a) FBP, 0.5 mm, 120 kVp, SD = 55; (b) AIDR, 0.5 mm, 120 kVp, SD = 75; and (C) AIDR, 0.5 mm, 120 kVp, SD = 115.

**Figure 4:**
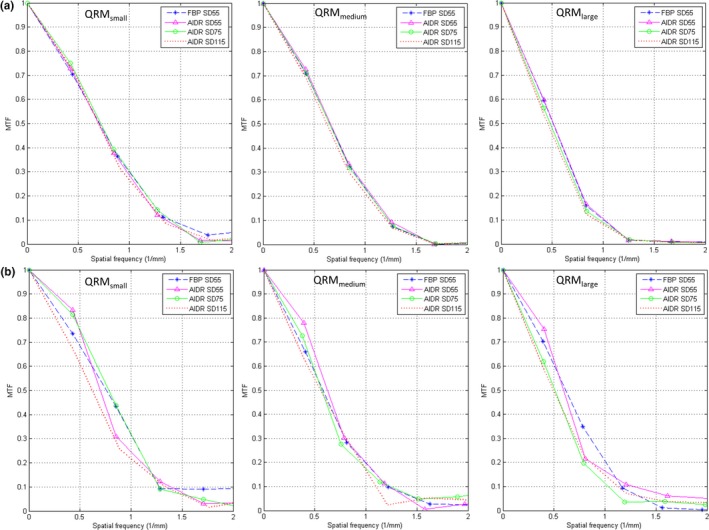
Comparison of MTF values from 120‐kVp CAC scans acquired with four different acquisition/reconstruction combinations for QRM_small_ (left), QRM_medium_ (mid), and QRM_large_ (right). ROI was placed over the boundaries (a) at soft tissue/lung interface and (b) between the large homogeneous insert with HA density of 200 mg/cm^3^ and the surrounding soft tissue.

Table 1 summarizes the CT numbers within background ROI of the 14 CAC protocols of various acquisition/reconstruction combinations. It was found that reducing slice thickness from 3 mm to 0.5 mm in FBP reconstruction results in fourfold increased dose in QRM_medium_ and QRM_large_ to achieve the same noise level (SD = 55). Due to the mA range in TCM, the CAC scan for QRM_small_ reconstructed at 3‐mm slice thickness using FBP was overexposed, so the image noise from 3‐mm FBP reconstruction is smaller than that from 0.5‐mm FBP reconstruction. Consequently, the CTDI_vol_ was increased by only 1.55‐fold when changing slice thickness from 3 mm to 0.5 mm in QRM_small_. For CAC scans acquired with 0.5‐mm slice thickness, all IR‐reconstructed images have lower image noise than FBP‐reconstructed images. Figure 2 demonstrates the CT images acquired with four different acquisition/reconstruction combinations for QRM_small_, QRM_medium_, and QRM_large_. It was found that the 1‐mm calcifications cannot be detected in some of the IR protocols (high SD value or large body size), so the total Agatston score for multivariate analysis was the sum over 3‐mm and 5‐mm calcifications. Figure 3. shows the histograms of calcifications with HA density of 200, 400, and 800 mg/cm^3^ in CAC scans acquired with three different acquisition/reconstruction combinations for QRM_medium_. A relative flattening of the calcification histogram was observed in CAC scans with higher image noise. Figure 4 compares the MTF values from 120‐kVp CAC scans acquired with four different acquisition/reconstruction combinations for QRM_small_, QRM_medium_, and QRM_large_, including (a) FBP, 0.5 mm, 120 kVp, SD = 55; (b) AIDR, 0.5 mm, 120 kVp, SD = 55; (c) AIDR, 0.5 mm, 120 kVp, SD = 75; and (d) AIDR, 0.5 mm, 120 kVp, SD = 115. It was found that increasing image noise or body size may degrade the MTF.

## 8 CAC SCORING

**Figure 5:**
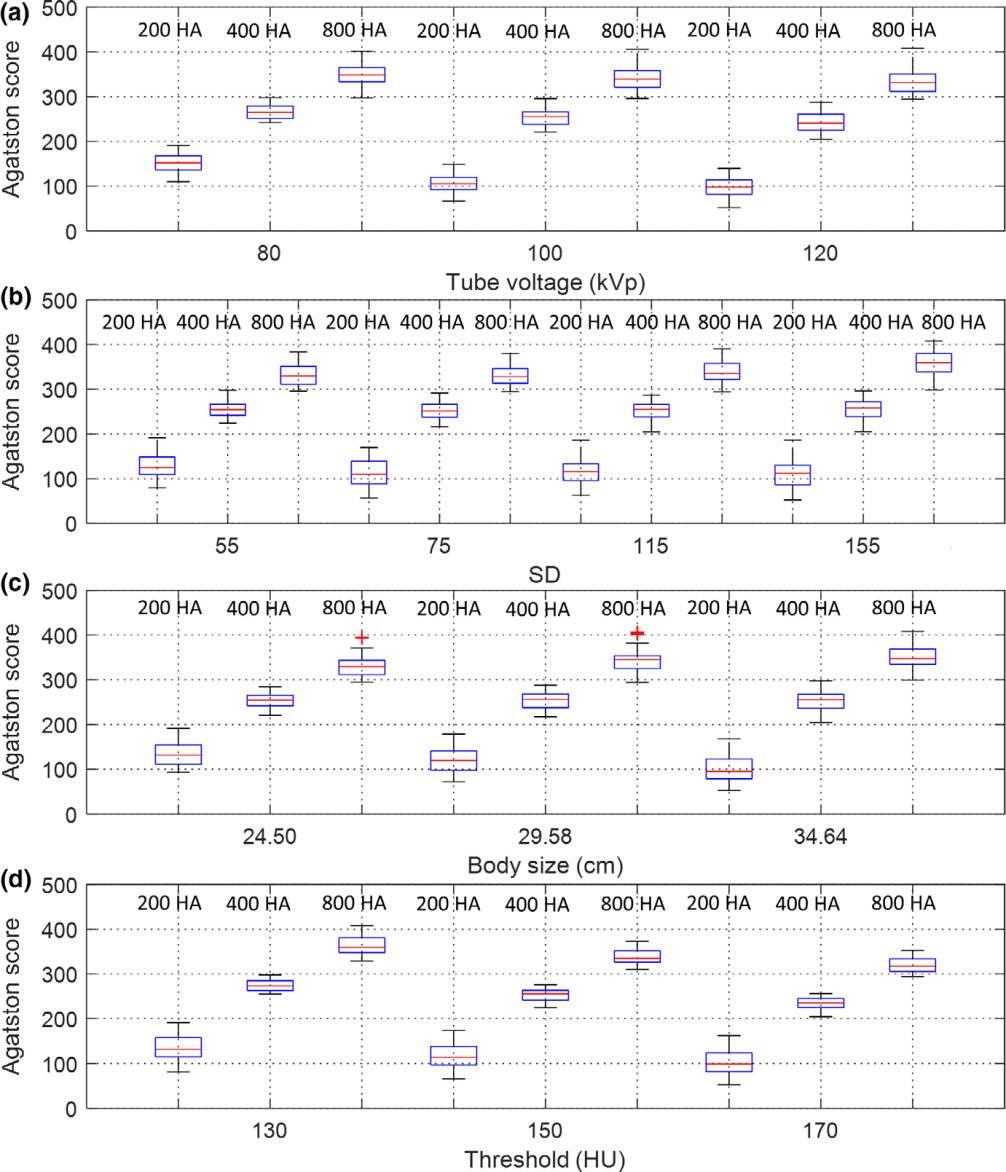
Box and whisker diagrams for Agatston score of calcification with HA density of 200, 400, and 800 mg/cm^3^ with respective to (a) tube voltage, (b) SD, (c) body size, and (d) HU threshold.

The box and whisker plots shown in Fig. 5 summarize the impact of tube voltage, SD, body size, and HU threshold on the total Agatston scores from 108 IR measurements (3 kVp values*4 SD values*3 phantom sizes*3 HU thresholds) for calcifications with HA density of 200, 400, and 800 mg/cm^3^. The red line in each box represents the median of the distribution, whereas the top and bottom of each box represent the 25^th^ and 75^th^ percentile of the distribution, respectively. The whiskers extend to the 99.3% confidence interval (±2.7 sigma). The asterisks are extreme outliers beyond the whiskers. For calcifications with different HA densities, similar tendency was found in [Fig. 5(a) and (d)]. but not in [Figs 5(b) or 5(c)].

## 9 MULTIVARIATE ANALYSIS

The results of regression analysis of Eq. (3) for the Agatston score of calcifications with HA density of 200, 400, and 800 mg/cm^3^ are shown in Table 2. It was found that all independent variables are statistically significant predictors of Agatston score, except the SD value and body size for AS_400HA_. For AS_200HA_, 1/kVp is the most significant predictor (β = 0.72), followed by body size (β = −0.43), 1/threshold (β = 0.41), and SD value (β = −0.16). The corresponding results for AS_400HA_ are 1/threshold (β = 0.79), 1/kVp (β = 0.47), SD value (β = 0.04), and body size (β = −0.02). As for AS_800HA_, the corresponding results are 1/threshold (β = 0.69), SD value (β = 0.40), body size (β = 0.33), and 1/kVp (β = 0.25). High multicollinearity was not observed among independent variables in the three AS models (VIF < 10). The regression equations that expresses the relationship between Agatston score and the predictors for calcifications with HA density of 200, 400, and 800 mg/cm^3^ are as follows:

(4) $${\text{AS}}_{200\text{HA}}=-25.14+13200.56\times \left(\frac{1}{\text{kVp}}\right)-0.13\times \left(\text{SD}\right)-3.28\times \left(\text{body}\;\text{size}\right)+17457.67\times \left(\frac{1}{\text{threshold}}\right)$$

(5) $${\text{AS}}_{400\text{HA}}=45.69+5683.61\times \left(\frac{1}{\text{kVp}}\right)+0.02\times \left(\text{SD}\right)-0.08\times \left(\text{body}\;\text{size}\right)+22171.06\times \left(\frac{1}{\text{threshold}}\right)$$

(6) $${\text{AS}}_{800\text{HA}}=45.60+3896.72\times \left(\frac{1}{\text{kVp}}\right)+0.28\times \left(\text{SD}\right)+2.08\times \left(\text{body}\;\text{size}\right)+24606.01\times \left(\frac{1}{\text{threshold}}\right)$$

**Table 2:** Statistical analysis results of the regression model for Agatston score of calcification with HA density of 200, 400, and 800 mg/cm^3^.

	B			β			t (p)			VIF		
	200 HA	400 HA	800 HA	200 HA	400 HA	800 HA	200 HA	400 HA	800 HA	200 HA	400 HA	800 HA
1/kVp	13200.56	5683.61	3895.72	0.72	0.47	0.25	23.16* (*P* < 0.01)	12.45* (*P* < 0.01)	5.92* (*P* < 0.01)	1.00	1.00	1.00
SD	−0.13	0.02	0.28	−0.16	0.04	0.40	−5.12* (*P* < 0.01)	1.15 (*P* = 0.23)	9.45* (*P* < 0.01)	1.00	1.00	1.00
Body size	−3.28	−0.08	2.08	−0.43	−0.02	0.33	−13.93* (*P* < 0.01)	−0.40 (*P* = 0.68)	7.63* (*P* < 0.01)	1.00	1.00	1.00
1/threshold	17457.67	22171.06	24606.01	0.41	0.79	0.69	13.26* (*P* < 0.01)	21.02* (*P* < 0.01)	16.17* (*P* < 0.01)	1.00	1.00	1.00

**Figure 6:**
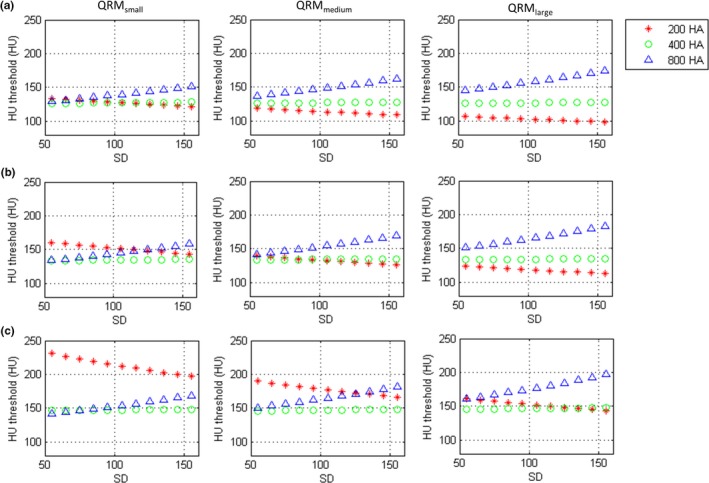
The HU thresholds as a function of SD estimated based on the regression models for (a) 120‐kVp, (b) 100‐kVp, and (c) 80‐kVp CAC scans reconstructed at 0.5‐mm slice thickness using AIDR 3D for three phantom sizes to reach the same Agatston score obtained from routine CAC scans.

The regression model in Eq. (4), (5), (6) yielded an R^2^ of 0.9005, 0.8531, and 0.8117, respectively. Figure 6 shows the HU thresholds as a function of SD estimated based on the regression models in Eq. (4), (5), (6) for 120‐, 100‐, and 80‐kVp CAC scans reconstructed at 0.5‐mm slice thickness using IR for three different phantom sizes to reach the same Agatston score obtained from routine CAC scans. These results indicate that the HU threshold should be adjusted according to the imaging condition, whereas a 130‐HU threshold is appropriate for 120‐kVp CAC scans acquired with SD = 55 for QRM_small_.

## 10 DISCUSSION

### 10.1 IR reconstruction in CAC scoring

Decades of prognostic data support the value of Agatston score for clinical risk predication. However, Agatston score uses 3‐mm slice thickness and a 130‐HU threshold for detecting calcium in FBP reconstruction. Implementation of the Agatston approach as the gold standard has, in some ways, slows the development and evaluation of alternative, optimized scan parameter.^5^ A reduced slice thickness may improve the accuracy in evaluating the actual presence of coronary calcium by decreasing the partial volume effects. The primary benefit would be the early detection of merging microcalcification, and a potential increase in total number of calcified lesions detected. However, thin‐slice reconstruction could be associated with higher image noise, which may bias the detection of calcification. IR allows substantial noise reduction in low‐dose CT, but it has been reported that IR techniques may underestimate CAC scores, particularly at low score ranges. Gephard et al.^25^ demonstrated a reduction in Agatston score when using the Adaptive Statistical Iterative Reconstruction (ASiR; GE Healthcare, Waukesha, USA) algorithm, and the reduction range is associated with the percentage of ASiR, ranged from 6.0% to 22.4% for 0–100% ASiR in Agatston score. Kurata et al. demonstrated a systematic decrease between 8% and 48% in Agatston score when using 10–50% of Sinogram‐affirmed Iterative Reconstruction (SAFIRE; Siemens Medical Solutions, Malvern, USA) blending.^26^ On the other hand, Rodrigues et al.^27^ and Blobel et al.^23^ showed good agreement in calcium scores between FBP and AIDR 3D protocols. Consistency in Agatston score between FBP and AIDR 3D was observed in our results acquired with 120 kVp and SD = 55 for QRM_small_, but not in those acquired with low‐dose protocols or larger phantoms. Overall, these studies indicate that CAC scores differ considerably by acquisition and reconstruction methods.

## 11 RELATIONSHIP BETWEEN IMAGE QUALITY AND AGATSTON SCORES

As seen in Fig. 5, the Agatston scores from 108 IR measurements increased as decreasing kVp, which can be explained by the increase in CT number of calcifications at lower tube voltage.^23^, ^24^ This phenomenon was observed in all calcification densities and was most obvious in the Agatston score of calcifications with HA density of 200 mg/cm^3^. With regards to the impact of SD value and body size, it was found that low tube current or high patient attenuation would lead to the decrease in calcium scores for less dense calcifications (eg, 200 mg/cm^3^), but this trend was reversed in dense calcifications (eg, 800 mg/cm^3^). According to Fig. 2 and 4, the spatial resolution of CAC scans was degraded by increased SD value or body size, which may be due to the increase in statistical fluctuation and scattered radiation. The degradation of spatial resolution would lead to the increase in partial volume artifacts, thus increasing the number of calcification pixels but with decreased intensity. For calcifications with HA density of 200 mg/cm^3^, the decrease in intensity due to partial volume effect may lead to CT numbers less than 130 HU in boundary pixels (Fig. 3), and truncation of these pixels would lower the Agatston score. As for calcifications with HA density of 800 mg/cm^3^, the CT numbers of boundary pixels were also decreased when increasing SD value or body size, but should still be larger than 400 HU (Fig. 3). Besides, the number of calcification pixels was increased due to partial volume effect, thus increasing the Agatston score. Regarding calcifications with HA density of 400 mg/cm^3^, it is suspected that the influences of low intensity (small w_i_) and more pixels (large A_i_) owing to partial volume effect on Agatston score cancelled each other out, so changing SD value or body size did not cause obvious variation in AS_400HA_. By definition, reducing CT number threshold would increase the Agatston score, and vice versa.^23^, ^24^ This phenomenon was also observed in our results, which was adapted to determine the optimal HU threshold for CAC scans undergoing various imaging conditions to ensure the quantification accuracy of calcium scoring from CAC scans reconstructed at 0.5‐mm slice thickness using IR relative to the conventional protocol.

## 12 OPTIMIZED HU THRESHOLDS BASED ON MULTIVARIATE ANALYSIS

In this work, a multiple linear regression method was used to analyze how the tube voltage, SD value, body size, and HU threshold affect the Agatston scores of 3 calcification densities for IR‐reconstructed CAC scans acquired with 0.5‐mm slice thickness simultaneously. Each regression relationship has R^2^ larger than 0.80, indicating a good fit to the data. Based on the regression models, the HU thresholds as a function of SD estimated to ensure the quantification accuracy of calcium scores for 120‐, 100‐, and 80‐kVp CAC scans reconstructed at 0.5‐mm slice thickness using IR for patients with body size of 24.50 cm (QRM_small_), 29.58 cm (QRM_medium_), and 34.64 cm (QRM_large_) were proposed (Fig. 6). Our results demonstrate that a 130‐HU threshold is appropriate for 120‐kVp CAC scans acquired with low SD values, performed on small‐sized patients. However, for calcifications with HA density of 200 mg/cm^3^, a lower HU threshold should be applied for 120‐kVp CAC scans acquired with higher SD value to prevent underestimation of Agatston score, especially for large‐sized patients. On the other hand, regarding calcifications with HA density of 800 mg/cm^3^, a higher HU threshold should be used to avoid overestimation of Agatston score. Once dose reduction is achieved by reducing tube voltage, a higher HU threshold should be applied to prevent overestimation of Agatston score, especially for small‐sized patients with less dense calcifications.

## 13 LIMITATIONS

Several limitations to this study need to be acknowledged. First, only a single manufacturer's CT system was investigated, so the proposed CAC scans and the corresponding HU threshold cannot be applied to CT systems from different manufacturers. However, the workflow to optimize data acquisition and quantification in this study may be used for AEC systems which modulate tube current according to a predefined image quality index, for example, SD in Canon and noise index (NI) in GE. Second, because it is not possible to evaluate the same individual with different body sizes, this work concerns a phantom study. It is possible that results differ under more extreme conditions, for example, denser calcification or larger body size. Additional studies assessing the optimization workflow for clinical patients on different CT systems will be needed and valuable.

## 14 CONCLUSION

Reduced slice thickness may contribute to the early detection of emerging microcalcification and a potential increase in the total number of calcified lesions detection. But there is an inherent tradeoff between slice thickness and image noise. Hence, this work evaluated the simultaneous influence of kVp, SD, body size, and HU threshold on calcium scoring for CAC scans that were reconstructed at 0.5‐mm slice thickness using IR through multivariate analysis. Regression results were used to optimize the HU threshold under various imaging conditions to calibrate the resulting Agatston scores to be consistent with those obtained from reference protocol. Our study results may provide a potential strategy to improve the reliability of calcium scoring by reducing partial volume effect while achieving the ALARA principle.

## CONFLICTS OF INTEREST

The authors have no relevant conflicts of interest to disclose.
